# Challenges to the Vestibular System in Space: How the Brain Responds and Adapts to Microgravity

**DOI:** 10.3389/fncir.2021.760313

**Published:** 2021-11-03

**Authors:** Jérome Carriot, Isabelle Mackrous, Kathleen E. Cullen

**Affiliations:** ^1^Department of Physiology, McGill University, Montreal, QC, Canada; ^2^Department of Biomedical Engineering, Johns Hopkins University, Baltimore, MD, United States

**Keywords:** afferent, brainstem, cerebellum, neural coding, vestibulo-ocular reflex, vestibulospinal reflex, self-motion

## Abstract

In the next century, flying civilians to space or humans to Mars will no longer be a subject of science fiction. The altered gravitational environment experienced during space flight, as well as that experienced following landing, results in impaired perceptual and motor performance—particularly in the first days of the new environmental challenge. Notably, the absence of gravity unloads the vestibular otolith organs such that they are no longer stimulated as they would be on earth. Understanding how the brain responds initially and then adapts to altered sensory input has important implications for understanding the inherent abilities as well as limitations of human performance. Space-based experiments have shown that altered gravity causes structural and functional changes at multiple stages of vestibular processing, spanning from the hair cells of its sensory organs to the Purkinje cells of the vestibular cerebellum. Furthermore, ground-based experiments have established the adaptive capacity of vestibular pathways and neural mechanism that likely underlie this adaptation. We review these studies and suggest that the brain likely uses two key strategies to adapt to changes in gravity: (i) the updating of a cerebellum-based internal model of the sensory consequences of gravity; and (ii) the re-weighting of extra-vestibular information as the vestibular system becomes less (i.e., entering microgravity) and then again more reliable (i.e., return to earth).

## Introduction

On earth, gravity is a force to which we are constantly exposed starting from the day we are born (Lacquaniti et al., 2014). During our everyday activities, it is vital that the brain accounts for the physical force of gravity (Honeine et al., 2013, 2014; Jansen et al., 2014). This is because, during our natural behaviors, gravity produces disequilibrium torques that must be counteracted by our motor systems to maintain balance and prevent falls. Accordingly, our feedforward compensatory pathways, postural strategies, and locomotor patterns all require taking gravity into account to ensure the maintenance of equilibrium during everyday activities—including quiet standing, arm reaching and locomotion (Cordo and Nashner, 1982; Papaxanthis et al., 1998; Sylos-Labini et al., 2013; Honeine et al., 2014; Lacquaniti et al., 2014; Macaluso et al., 2017). Moreover, the physical force of gravity provides a vital world-based reference to which we can anchor our perception of spatial orientation as well as control of balance (Lackner and DiZio, 2005; Lacquaniti et al., 2014; Panic et al., 2015).

Importantly, during space exploration missions, the force of gravity becomes minimal. As a result, nearly 70% of all astronauts experience impaired balance, locomotion, gaze control, dynamic visual acuity, eye–head–hand coordination, and/or motion sickness within the first 3–4 days of both space flight and then again after returning to earth (Lackner and Dizio, 2006; Souvestre et al., 2008). These symptoms arise because changes in gravity alter the sensory input from the vestibular system, which in turn generates a persistent conflict (i.e., mismatch) between expected and actual sensory vestibular inputs during active movements (Oman and Cullen, 2014). Then, in the days following such changes in gravity, astronauts show sensorimotor adaptation that results in improved motor performance. As discussed below, recent experiments using ground-based models have furthered our understanding of the neural mechanisms that underlie sensorimotor adaptation and thus have important implications regarding the interpretation of the results from flight-based studies. Notably, these studies have established the neural mechanisms that underlie the brain’s computation of an estimate of gravity and self-motion during active behaviors and have also provided evidence for the re-weighting of vestibular inputs in conditions where it becomes less reliable. In this review, we consider these findings in the context of experiments that have studied the neurovestibular adaptation during and after space flight and the implications for improving human performance during and following space exploration.

## Gravity Is Important on Earth: Posture, Perception, and Behavior

The findings of theoretical as well as behavioral studies have led to the longstanding hypothesis that the brain builds an internal model of the expected sensory consequence of our own actions (Wolpert et al., 1998; Wolpert and Ghahramani, 2000). During self-motion, this internal model is required for the maintenance of posture, accurate spatial orientation, and the generation of precise voluntary movement (Cullen, 2019). Specifically, by comparing the incoming sensory information from different modalities (i.e., vestibular signals with information from the proprioceptive, somatosensory, and visual systems) with that predicted by its internal model, the brain anticipates and validates the consequences of the force of gravity (McIntyre et al., 1998; Zupan et al., 2002). On earth, the expectation of the constant force of gravity is an inherent component of this internal model. However, during space exploration, the force of gravity becomes negligible resulting in a mismatch between the brain’s expectation of the sensory consequences of movement and that which is actually experienced due to the resultant unloading of the otoliths. This mismatch has important implications for astronauts, during and after space flight, since it results in impaired behavioral performance in the first days of exposure to an altered gravity environment. However, after 1–5 days of space flight and ~1 week after landing these symptoms largely disappear, implying that the brain has adapted to the new gravitational environment.

### Posture and Locomotion in Space

The maintenance of upright posture during quiet standing requires overcoming the force of gravity. The biomechanics of human posture can be well modeled by an inverse pendulum (Winter et al., 1997, 1998). Accordingly, we constantly oscillate around an equilibrium point and small corrective movements are required to prevent falling. The neural mechanisms that stabilize upright posture in 1 *g* generally persist when initially exposed to microgravity, even though they are no longer necessary (Clément et al., 1984; Mouchnino et al., 1996; Massion et al., 1997; Vernazza-Martin et al., 2000; Baroni et al., 2001). Indeed, the human body naturally assumes a more neutral posture in microgravity characterized by a semi-crouched torso, flexed arms and legs, and forward bent neck and head (Andreoni et al., 2000; Han Kim et al., 2019). While the brain’s internal model of postural control appears structurally stable in the short-term, it remains unknown whether the neural mechanisms that stabilize the upright posture in 1 *g* continue to operate during long missions in space. However, it appears likely that this is not the case. Indeed, postural stability is often related to the Hoffman reflex, an otolith-spinal reflex (Chen and Zhou, 2011 for review). Muscle activity associated with the Hoffman reflex has been shown to reach low values with longer delays after 7 days in space (Reschke et al., 1984; Watt et al., 1986). It appears that in microgravity, the information coming from the otolith organs to the motoneurons is gradually reinterpreted. Similarly, following re-entry into 1 *g*, the neural mechanisms that stabilize the upright posture appear to be largely disrupted following both short (1–2 weeks) and long (4–6 month) duration spaceflight (Jain et al., 2010; Wood et al., 2015). Interestingly, more severe and persistent deficits occur in the latter case. Rapid recovery is reported on the first day after return, with more gradual improvement in the following weeks ultimately returning performance to pre-flight levels (Paloski et al., 1992; Reschke et al., 1998).

In addition to postural instabilities, astronauts often experience oscillopsia during locomotion following space flight, suggesting that head-trunk coordination is impaired (Bloomberg et al., 1997). Specifically, the coherence between pitch head and vertical trunk movements is reduced following space flight (Bloomberg et al., 1997; Mulavara et al., 2012) similar to what has been observed in patients with altered vestibular input due to peripheral vestibular loss (Mulavara et al., 2012) or the application of galvanic vestibular stimulation (Moore et al., 2006). An interesting fact is that head-trunk coordination is better during locomotion after re-entry in more experienced astronauts (e.g., number of flights; Bloomberg et al., 1997; Moore et al., 2006) suggest that experience influences the ability to rapidly update a vestibular based internal model for the control of posture and locomotion.

### Perception in Space

During space flight, astronauts also report spatial disorientation and destabilizing sensations. On earth, many aspects of our environment, including ourselves, are “gravitationally polarized.” The brain continually computes our head and body orientation relative to gravity, using vestibular and other sensory information (reviewed in Goldberg et al., 2012). Spaceflight violates many of the regularities that characterize our orientation on the ground (Lackner and DiZio, 2000). For example, due to the lack of otolith input that normally signals head orientation relative to gravity, astronauts can lose all sense of spatial anchoring to their surroundings when their eyes are closed (Lackner and Graybiel, 1979). When their eyes are open, astronauts may intellectually know their position in relation to their surroundings, but they do not experience a normal sense of orientation with respect to the environment (Lackner and DiZio, 2000). As a result, sensations of inversion, tilt, and virtually every combination of body orientation and vehicle orientation have been reported. With increased flight duration such illusions, which can be experienced immediately on transition into microgravity, tend to abate as astronauts adapt to their new environment (Lackner and DiZio, 2000).

Perceptual adaptation to altered gravity has also been studied using centrifugation on the ground as well as in space (e.g., Clément et al., 2001). For example, shortly following transition into microgravity, subjects experience a roll tilt illusion during centrifugation that is similar to that observed during ground-based experiments (~45°). However, during prolonged exposure (i.e., 16 days in microgravity), the illusion of tilt increased, such that subjects reported that they felt as if they were lying on their side (~90°). These results suggest that the brain initially continues to use its ground-based model of the sensory consequences of gravity during early space flight, which it then adapts to account for the new microgravity environment. Likewise, perceptual adaptation was evidenced by larger tilt illusion values upon re-entry compared to the pre-flight values. It is important to note that impaired perception during gravitational transition compromises an astronaut’s ability to control the spacecraft itself (Clément, 2018). For instance, it has been reported that astronauts that failed to land safely had episodes of spatial disorientation during the procedure (Clark and Bacal, 2008). These pilots showed vestibular dysfunction that was correlated with their performance in controlling the spacecraft during the landing procedure.

### Voluntary Movement in Space

Finally, there is accumulating evidence that the accurate control of voluntary movements, such as reaching, is correspondingly altered during space flight (Carriot et al., 2004; Scotto Di Cesare et al., 2014; Gaveau et al., 2016; White et al., 2020). When instructed to reach up or down, human subjects demonstrate asymmetric arm kinematic suggesting that the brain also uses an internal model of gravity to predict and take advantage of its mechanical properties to optimize effort (Gaveau et al., 2016). Interestingly, this asymmetry disappears in microgravity (Carriot et al., 2004; Crevecoeur et al., 2010; Gaveau et al., 2016). Additionally, pointing accuracy drastically decreases in absence of gravity (Carriot et al., 2004). While it was initially proposed that this occurs due to the reduction of the arm weight in microgravity (Bringoux et al., 2012), a subsequent EEG study reported increased activity within the vestibular network during a comparable visuo-motor task (Cebolla et al., 2016). Moreover, comparable effects have been reported in an ground-based model when vestibular input was ablated *via* labyrinthectomy (Angelaki, 2021). Thus, taken together, the altered vestibular inputs experienced during space flight likely contribute not only to the observed impairments in postural and perceptual performance but also to changes in the kinematics and accuracy of voluntary movements.

It is noteworthy that to date, most studies of voluntary movements in microgravity have been performed during parabolic flights and thus it was not possible to investigate long-term adaptation. However, the findings of ground-based centrifugation experiments have shown that reaching patterns can rapidly adapt to new force field environments when tested (i.e., 10–15 movements; Lackner and Dizio, 1994). Moreover, evidence from studies of astronauts following re-entry is consistent with rapid adaptation. Specifically, sensorimotor learning (Mulavara et al., 2010), as well as eye–head and head-trunk coordination (Glasauer et al., 1995; Bloomberg et al., 1997; Reschke et al., 1998; Bloomberg and Mulavara, 2003; Courtine and Pozzo, 2004; Clément and Wood, 2014) recover rapidly in the first day after return from short- and long-term missions, with an improvement that is more gradual in the following weeks. Thus, it seems likely that the brain likewise adapts its control of voluntary movements over the long-term in microgravity.

### Conclusions

Overall, the absence of gravity severely impairs motor and perceptual performance. Although the computation of gravity relies on the integration of sensory information from our different senses, the role of the vestibular signal appeared to be omnipresent in most if not all human behaviors in space. At this stage, it is thus fundamental to understand how the gravity signal is computed from vestibular inputs.

## Our Brains Are Wired to Keep Track of Gravity: What Happens to The Vestibular System in Space

### Vestibular Sensory Organs and Peripheral Transmission

**Figure 1:**
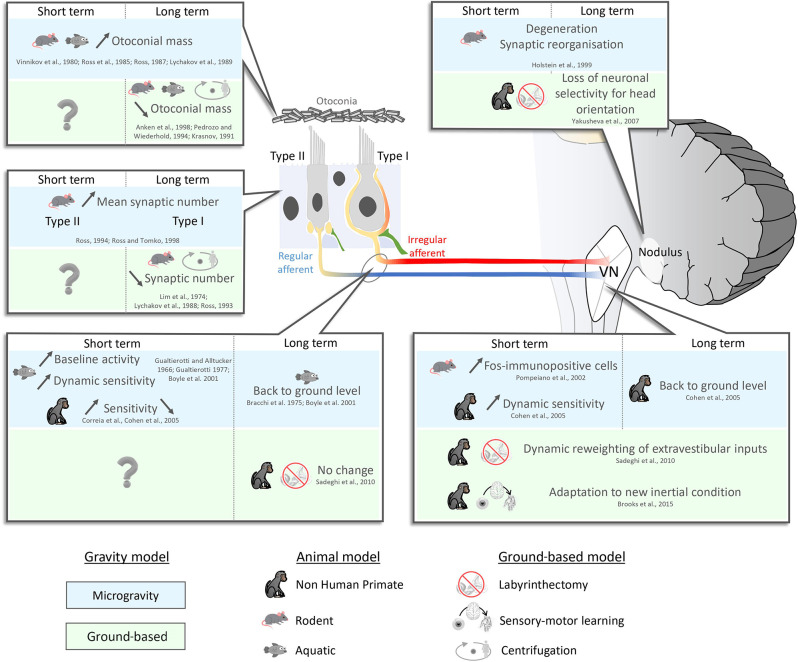
Neurophysiological short- (<7 days) and long (>7 days) term- adaptation to microgravity and ground-based model. *Upper left panel*: the mass of the otoconia increases following short-term exposure to microgravity (Vinnikov Ia et al., 1980; Ross et al., 1985; Lychakov et al., 1989). This increase in mass is assumed to be maintained as long as there is no change in the gravitational environment. The opposite phenomenon occurs in hypergravity (decrease in mass; Krasnov, 1991; Pedrozo and Wiederhold, 1994; Anken et al., 1998; reviewed in Cohen et al., 2005). To date, however, the time course is unknown since testing was only done after long-term centrifugation (>24 days). *Middle left panel*: After 7–9 days in microgravity, type II vestibular hair cells increase in size and number (Ross, 1993, 1994, 2000). Then after 2 weeks in microgravity, the number of type I cells increases as well (Ross and Tomko, 1998). In hypergravity, only type II hair cells show a significant decrease in number (e.g., following 14–30 days of centrifugation; Lychakov et al., 1989; Ross, 1993). *Bottom left panel*: Immediately after entering microgravity, studies across species have reported increases in vestibular afferent baseline activity and sensitivity (Gualtierotti and Alltucker, 1967; Gualtierotti, 1977; Boyle et al., 2001). However, when considered alone, studies in NHPs have been inconclusive as some report increases and other report decreases in sensitivity (Correia et al., 1992; Cohen et al., 2005). After 5 days in microgravity, vestibular afferent responses return to ground levels (Bracchi et al., 1975; Boyle et al., 2001). On earth, 1 month after complete unilateral vestibular lesion, afferent responses in the intact nerve remain comparable to control levels (Sadeghi et al., 2007, 2010, 2011, 2012). *Bottom right panel*: Across animal models, the sensitivities of vestibular nuclei neurons initially increase in microgravity and then return to baseline levels after a week (Pompeiano et al., 2002; Cohen et al., 2005). On earth, following labyrinthectomy, vestibular nuclei neurons that normally only respond to vestibular input before lesion, show the emergence of responses to extravestibular inputs (efference copy, proprioception) after lesion (Sadeghi et al., 2007, 2010, 2011, 2012). During sensory-motor adaptation, vestibular neurons update their response to altered sensory feedback (Brooks and Cullen, 2014; Brooks et al., 2015; Mackrous et al., 2019). *Top right panel*: The cerebellum displays synaptic reorganization as early as 24 h following the transition to microgravity, which remains for at least 18 days (Holstein et al., 1999). On earth, following labyrinthectomy, cerebellar neurons lose their ability to discriminate between tilt and translation (Yakusheva et al., 2007).

To date, many investigators have studied how the vestibular system responds and adapts to the transition from gravity to microgravity (Figure 1). As reviewed above, the absence of gravity leads to unloading of the otoliths such that they are no longer stimulated as they would be on earth by changes in the head’s spatial orientation. Early experiments in rats and frogs suggested that this unloading causes an increase in the mass of the otoconia (i.e., the small crystals of calcium carbonate which couple mechanic forces to the activation of sensory hair cells in the utricle and saccule) following short-term (i.e., 7 days) exposure to microgravity (Vinnikov Ia et al., 1980; Ross et al., 1985, 1987; Lychakov et al., 1989). Correspondingly, experiments in model systems have shown that the opposite phenomenon appears to occur in hypergravity (cichlid fish: Anken et al., 1998; marine mollusk larvae: Pedrozo and Wiederhold, 1994; rats: Krasnov, 1991; reviewed in Cohen et al., 2005). Most recently, Boyle and Varelas (2021) investigated the structural remodeling that occurs in the otoconia of mice using electron microscopy. Interestingly, these investigators found evidence for a mass addition to the otoconia outer shell, following exposure to long but not short duration spaceflight (or hindlimb unloading), as well as the thinning of the inner shell and cavitation of the otoconia following centrifugation. Likewise, structural changes following hindlimb unloading have been reported following long but not short duration hindlimb unloading (i.e., 90 days, Boyle and Varelas, 2021) vs. 160 days (Zhang et al., 2005). Accordingly, taken together these findings suggest that the otoconial mass adapts to fluctuations in the gravitational stimulus to maintain a consistent force on the maculae in astronauts during space flight. Future work will be required to fully understand the detailed time course of these changes.

The vestibular receptor cells in all mammalian end organs, including the otoliths, are called hair cells and are divided into two subtypes (Figure 1, left panel). These subtypes, termed type I and type II hair cells, occur in nearly equal ratios. Type I hair cells are defined by the presence of calyceal afferent innervation, while in contrast Type II hair cells synapse upon discrete bouton afferent terminals (Figure 1, left panel; reviewed in Cullen, 2019). Intriguingly, prolonged exposure to microgravity (>7 days) also appears to primarily impact the structure of type II hair cells (Ross, 1993, 1994, 2000). For example, ultrastructural analysis has demonstrated statistically significant increases in the number of type II utricular hair cell synapses in mice after a 9-day space flight (Ross, 1994). After 2 weeks in microgravity, an increase in the mean number of presynaptic processes ending on the calyces of type I cells has also been reported (40%; Ross and Tomko, 1998). A more recent study interestingly reported a reduction, rather than increase, in the synapse densities of the hair cells in the mouse utricle following 15 days of exposure to microgravity (Sultemeier et al., 2017). While differences in the approach used by Ross et al. (1985; electron microscopy) vs. Sultemeier et al. (2017; immunohistochemistry) complicate direct comparison, taken together the results of these studies suggest that vestibular hair cells, at least in rodents, can demonstrate adaptative changes in response to altered gravity. These peripheral adaptative changes, combined with those occurring at subsequent stages of vestibular processing (detailed below), likely contribute to the changes in utricular function that have been reported in astronauts immediately after returning from space flight (Hallgren et al., 2016; Reschke et al., 2018).

Finally, following exposure to microgravity, changes have also been reported at the next stage of peripheral vestibular processing, namely in the vestibular nerve afferents. Initially after entering microgravity both the baseline activities and sensitivities of otolith afferents substantially increase (reviewed in Clément et al., 2020). This finding is consistent across all non-mammalian animal models that have been tested (toadfish: Boyle et al., 2001; bullfrog: Gualtierotti and Alltucker, 1967; Gualtierotti and Bailey, 1968; Bracchi et al., 1975; Gualtierotti, 1977). Otolith afferent baseline activities and sensitivities then appear to return to control levels after ~5 days (Bracchi et al., 1975) and/or 24 h after returning to the ground (Boyle et al., 2001). It has been proposed that this initial hypersensitivity of otolith afferents induced by microgravity is due to presynaptic adjustment of synaptic strength in the hair cells reviewed above (Ross, 2000).

To date it remains unknown whether vestibular afferent sensitives likewise change during the first days of space flight in mammals. Afferent recordings were made in monkeys after 12 and 14 days of flights during two COSMOS missions (COSMOS 2044 and COSMOS 2229, respectively). However, these two missions reported contradictory findings (increased vs. decreased gains relative to pre-flight levels; Correia et al., 1992; Cohen et al., 2005). Indeed, given the inherent variability of monkey afferent response gains (Sadeghi et al., 2007; Massot et al., 2011; Jamali et al., 2019), the low numbers of afferents recorded in each study were likely not sufficient to make a pre-post flight comparison. In this context, it is important to note that the vestibular efferent system does not appear to play a significant role in the short-term adaptation of afferent coding in mammals as it does in lower vertebrate species (reviewed in Cullen and Wei, 2021). Thus, how microgravity influences the responses of vestibular afferents in mammals remains an open question.

### Central Vestibular Processing

Vestibular afferents target neurons in the vestibular nuclei comprise the first stage of central vestibular processing. Most vestibular nuclei neurons integrate inputs from both otolith and canal afferents (reviewed in Goldberg et al., 2012; Cullen, 2019). Single unit recordings have been made from the vestibular nuclei of rhesus monkeys on several Russian “Cosmos/Bion” Missions between the Bion 6 (Cosmos 1514) through Bion 11 projects. The findings of these studies are detailed in “Final Reports” submitted by investigators to Russia’s Institute of Biomedical Problem, as well as in some published reports (reviewed in Cohen et al., 2005: Sirota et al., 1984, 1986, 1987, 1988a,b, 1989a,b, 1990a,b,c, 1991a,b,c; Shipov et al., 1986; Sirota, 1988; Kozlovskaya et al., 1989, 1991, 1994; Yakushin et al., 1989, 1990, 1992; Badakva et al., 1993). Overall, investigators reported increases in the sensitivities of vestibular nuclei neurons to both linear and rotational head motion during the first days of space flight, with a subsequent return to normal (reviewed in Cohen et al., 2005). This finding was surprising given that microgravity affects the linear forces sensed by the otolith but not the rotations sensed by the canals (Cohen et al., 2005). Notably, neural sensitivities to linear head motion reached a maximum by the end of the first week in space while neural sensitivities to rotational head motion increased only within the first days of flight and then returned to normal levels within this same time frame.

The effect of microgravity on vestibular nuclei activity has also been studied by quantifying the expression of the early gene c-fos, which is a neural activity marker. For example, in ground-based models, galvanic stimulation and centripetal acceleration lead to increases in Fos immunoreactivity in the vestibular nuclei (Kaufman et al., 1992a,b; Kaufman and Perachio, 1994). Experiments done in space have likewise reported increased Fos expression in the vestibular nuclei of rats (particularly the medial and descending vestibular nuclei) 24 h postlaunch. Increased Fos expression has also been observed following return from a 17-day mission (Pompeiano et al., 2002). In contrast, Fos expression levels were comparable to control levels 13 days postlaunch and at 13 days postlanding, consistent with adaption occurring over time in response to altered gravity. Interestingly, while Fos expression was unchanged in autonomic regions that have been linked to motion sickness postlaunch (i.e., area postrema and nucleus tractus solitarius), significant increases were observed in these areas 24 h after landing (Pompeiano et al., 2004).

The vestibular afferents and vestibular nuclei both send direct projections to the caudal vermis of the cerebellum. In particular, the cerebellar nodulus receives significant input from vestibular otolith afferents. Ultrastructural changes in Purkinje cell dendritic morphology and/or the synaptic organization of their mossy fiber inputs have been reported when measured during 5–18 days of space flight (Krasnov and D’iachkova, 1986; Krasnov and Dyachkova, 1990). Similar changes are observed in nodular mossy fiber terminals. Additionally, major changes occur in the Purkinje cell cytoplasm within 24 h, including enlargement of the cisterns of the smooth endoplasmic reticulum, formation of long, stacked lamellar bodies, and the presence of degeneration (Holstein et al., 1999). Based on the last of these structural alterations, it has been proposed that excitotoxicity may play a role in the short-term changes in neural responses that are observed during space flight (Cohen et al., 2005).

Taken together, the findings of space-based experiments in central pathways demonstrate that the loss of otolith loading in microgravity (or reestablishment of loading following retry) leads to an increase in the sensitivity of vestibular pathways followed by adaptation over time. Below we consider the implications of ground-based research for providing insight into the neural mechanisms that underlie the sensorimotor adaptation required to ensure postural and perceptual stability, as well as the ability to generate accurate movements after exposure to altered gravity.

## What Are The Implications of Ground-Based Models for Understanding How The Vestibular System Adapts to Microgravity

### The Vestibular Cerebellum and Computation of Head Orientation Relative to Gravity

As reviewed in “Gravity Is Important on Earth: Posture, Perception, and Behavior” section above, there is consensus that the brain builds an internal model of the expected sensory consequence of our own actions (Wolpert et al., 1998; Wolpert and Ghahramani, 2000). During self-motion, this internal model is required for the maintenance of posture, accurate spatial orientation, and the generation of precise voluntary movement. The otoliths, like any inertial sensor (i.e., accelerometer), cannot distinguish forces produced by changes in the head’s orientation relative to gravity from those produced during translational self-motion. Thus, to compute a real time estimate of the head’s orientation relative to gravity on earth, the brain integrates rotational head motion information from the semicircular canals with otolith signals (reviewed in Goldberg et al., 2012). Ground-based single-unit recording experiments in head-restrained monkeys have shown that some Purkinje cells in the nodulus/uvula of the caudal vermis integrate otolith and semicircular canal inputs during passively applied self-motion to provide an estimate of current head orientation relative to gravity (reviewed in Angelaki and Cullen, 2008). Further, with the loss of canal input (i.e., *via* canal plugging) these same neurons lose their ability to discriminate between changes in the head’s orientation relative to the gravity and linear head acceleration (Yakusheva et al., 2007). This finding has led to the proposal that, on earth, the vestibular cerebellum computes internal models of the physical laws of motion to provide an estimate of the head’s orientation relative to gravity (reviewed in Goldberg et al., 2012).

### Cerebellar Prediction of the Dynamic Sensory Consequences of Gravity During Active Motion

More recently, single-unit recording experiments have further demonstrated that cerebellum-based mechanisms cancel the sensory consequences of gravity during active head movements (Mackrous et al., 2019). The activity of individual cerebellar output neurons was recorded while monkeys actively reoriented their heads relative to gravity. Strikingly, the robust vestibular responses displayed by neurons to the passive head motion were canceled during comparable active head movements. Indeed, such cancellation is required to maintain accurate postural control and perceptual stability. For example, on earth, vestibulo-spinal reflexes are vital to ensuring postural stability in response to unexpected changes in the head’s orientation; they send compensatory motor commands to the neck and axial/appendicular muscles that stabilize posture relative to space. However, when the same head motion is actively generated, these compensatory reflex responses are counterproductive because they would oppose the intended voluntary behavior through space. Indeed, during active movements, this cerebellum-based mechanism suppresses vestibulo-spinal reflex pathways during active movement relative to gravity (Mackrous et al., 2019).

It then follows that in space flight, following the transition to microgravity, both active and passive head movements will produce different (i.e., reduced) otolith afferent input compared to what they would produce on the ground. Notably, head tilts will continue to activate semi-circular canal but not otolith afferents. Thus, the brain experiences a mismatch between its expectation (internal model) of the resulting sensory feedback and actual sensory feedback that is experienced during head movements. During active movements, the initial mismatch between expected and actual otolith input will likely result in higher modulation in neurons in the vestibular nuclei as compared to earth. Over time, however, we speculate that cerebellum-based mechanisms underlie the ability to adapt to such mismatches and update the brain’s internal model to account for the new relationship between expected and actual sensory vestibular input that exists in microgravity.

Indeed, to date, such cerebellum-based adaptation has been demonstrated in ground-based experiments where a resistive load was applied to a monkey’s head while it generated voluntary head movements (e.g., Brooks et al., 2015). Because the application of the load initially altered the relationship between the motor command to move the head and its actual movement, the resultant vestibular sensory feedback was less than expected. Thus, initially, there was a mismatch and vestibular responses were not canceled during active head movements. However, cerebellar output neurons then show trial-by-trial adaptation to the new sensorimotor constraints after many active head movements—until there was again a match between the expected and actual sensory feedback. Once the internal model was updated and there was a match between actual and expected sensory feedback, vestibular responses were again canceled during active head movements. This finding has direct implications for behavior, since these cerebellar output neurons send descending projections to vestibular nuclei neurons that mediate vestibulo-spinal reflexes. Given that these reflexes are essential for maintaining posture and balance, the brain’s ability to adapt its descending commands to account for changes in the environment is essential. We propose that a similar cerebellum-based mechanism accounts for adapting to learning a new match between expected and actual sensory consequences of gravity when an astronaut is initially exposed to microgravity or returns to the ground following sustained exposure to microgravity.

Additionally, over longer time periods in microgravity, vestibular pathways are also likely to reweight this extra-vestibular information to compute more reliable estimates of head orientation. Prior ground-based studies in monkeys following peripheral vestibular loss have insights into the neural mechanisms that underlie the re-weighting of sensory information when vestibular information becomes less reliable (Sadeghi et al., 2010, 2011, 2012; Jamali et al., 2014). Notably, in normal rhesus monkeys (and presumably humans), central pathways do not integrate vestibular and proprioceptive signals at the level of the vestibular nuclei; vestibular nuclei neurons are insensitive to passively applied stimulation of proprioceptors. Instead, the integration of vestibular and proprioceptive only occurs at the next levels of vestibular processing, for example in the rostral fastigial nucleus of the cerebellum (Brooks and Cullen, 2009; Brooks et al., 2015) and vestibular thalamus (Marlinski and McCrea, 2008; Dale and Cullen, 2015). Surprisingly, however, following a peripheral vestibular loss, vestibular nuclei neurons demonstrate strong responses to passively applied stimulation of proprioceptors, suggesting that a form of homeostatic plasticity compensates for the reduced reliability of the vestibular input (Sadeghi et al., 2010, 2011, 2012).

Thus, the dynamic re-weighting of inputs from different modalities (i.e., extravestibular vs. vestibular) is observed even at the first stage of central processing in the vestibular nuclei following a peripheral vestibular loss. At least two types of extravestibular inputs substitute for the lost vestibular input: (1) proprioception; and (2) motor efference copy signals. Initially, robust responses to passive stimulation of neck proprioceptors are rapidly unmasked (within 24 h) and are linked to the compensation process as evidenced by faster and more substantial recovery of the resting discharge in proprioceptive-sensitive neurons (Sadeghi et al., 2010). Over the long term, efference copy signals also contribute to neuronal responses such that the efficacy of vestibular pathways is enhanced for active vs. passive self-motion (Sadeghi et al., 2010, 2011, 2012). Such re-weighting of extra-vestibular information in early vestibular pathways is also likely to occur in microgravity, where otolith organs are unloaded and thus are no longer stimulated as they would be on earth. We speculate that these results have implications for better understanding compensation and adaptation to vestibular functional disruption. Consistent with this proposal, recent MRI studies in astronauts pre- vs. post-flight have provided evidence for vestibular/proprioceptive sensory re-weighting and adaptive neuroplasticity at higher levels of processing in the cortex (Hupfeld et al., 2021).

Indeed, there is evidence from both space- and ground-based studies that such extra-vestibular sensorimotor feedback can rapidly influence the online processing of vestibular information for the control of balance (Marsden et al., 2003) and locomotion (Mulavara et al., 2012; Forbes et al., 2017). Additionally, we note that vision provides important “extra-vestibular” information about self-motion and spatial orientation, as was elegantly demonstrated by the “visual reorientation illusion” experiments of Howard and colleagues (e.g., Howard and Hu, 2001; Jenkin et al., 2003). Future experiments focused on the neural mechanisms responsible for the reweighing of extra-vestibular and vestibular information following exposure to altered gravity are required to fully understand the mechanisms responsible for the changes observed in astronaut performance/strategies during space flight.

In summary, we propose that the results of recent ground-based studies of the neural mechanisms underlying sensorimotor adaptation provide important insights into the central changes that occur in the brains of astronauts before and after space exploration missions.

## Connecting The Dots Between Ground- and Space-Based Neurophysiological Studies of Vestibular Pathways and Their Compensation

Above, we reviewed the results of space-based research that have revealed significant modifications in the cellular and subcellular structure of the vestibular pathways at multiple levels (Figure 1)—from the vestibular periphery (increase in the mass of the otoconia, initial hypersensitivity of otolith afferents) to the cerebellum (changes in Purkinje cell dendritic/synaptic morphology). On earth, two key ground-based models: (i) vestibular peripheral lesion (e.g., labyrinthectomy); and (ii) sensorimotor adaptation have proven essential to our fundamental understanding of the adaptive capacity of vestibular pathways and neural mechanisms that underlie this adaptation.

First, the results of ground-based single unit studies using vestibular peripheral lesions have been essential to furthering our knowledge of how central mechanisms compensate for the sustained reduction in vestibular input. Following unilateral labyrinthectomy, peripheral afferent responses in the intact nerve (i.e., the contralesional nerve) are comparable to control levels when recorded >1 month after lesion (Sadeghi et al., 2007). As reviewed in “What Are the Implications of Ground-Based Models for Understanding How the Vestibular System Adapts to Microgravity” section above, long-term compensation following vestibular peripheral lesion is mediated by “central strategies” based on the re-weighting of extra-vestibular inputs and updating of internal models. Likewise, as reviewed in “Our Brains Are Wired to Keep Track of Gravity: What Happens to the Vestibular System in Space” section above, in nonmammalian species peripheral afferent responses are comparable to control levels after 5 days in space (Bracchi et al., 1975) and 24 h after returning to the ground (Boyle et al., 2001). Thus, this latter result in nonmammalian species is similar to what has been observed in the intact nerve following unilateral labyrinthectomy in ground-based studies of primates. Nevertheless, future studies will be required to establish whether and how the sensitivities of mammalian vestibular afferent sensitives initially change during the first days of space flight.

Second, the findings of ground-based single unit studies of sensorimotor adaptation have provided additional insights into the adaptive capacity of central vestibular pathways that have important implications for space flight. As reviewed above, central vestibular neurons and cerebellar output neurons demonstrated trial-by-trial updating of altered sensory feedback during active head rotations (Brooks et al., 2015; Cullen and Brooks, 2015). These experiments establish the neural correlate for a cerebellum-based forward model that computes an estimate of the sensory consequences of voluntary motion (Brooks et al., 2015). Additionally, recent experiments have further made the important discovery that this forward model also continually accounts for the sensory consequences of gravity during active motion (Mackrous et al., 2019). Thus we predict that, during space flight and then again following landing, these same cerebellar output neurons show comparable updating to account for changes in the force of gravity.

Together, these findings suggest that the design of more effective countermeasures to maintain crew health and performance could be obtained by optimizing exercises that accelerate these early stages of compensation (i.e., sensory re-weighting and the updating of internal models). Further, reports of improved posture and locomotion after re-entry for more experienced astronauts (e.g., number flights; Bloomberg et al., 1997; Moore et al., 2006) suggest that experience influences the ability to rapidly update a vestibular based internal model and has interesting implications for the design of pre-flight training regimes. Future work coupling neuronal recordings with vestibular peripheral lesion and sensorimotor adaptation as well as other established ground-based models, such as centrifugation, will likely provide additional insight into the neural mechanisms that underlie and potentially facilitate adaptation during space flight.

## Conclusions

Most astronauts experience motor and perceptual impairments as well as motion sickness during the first 3–4 days of both space flight and then again after returning to earth (Lackner and Dizio, 2006). As we reviewed above, there are many reasons to believe that such symptoms occur due to a mismatch between the brain’s internal model of the expected sensory consequences of active behaviors and the actual sensory reafference that is experienced. Most notably, changes in the force of gravity alter the sensory input from the vestibular system, because the absence of gravity results in an unloading of the otoliths during space exploration, and then the reloading of the otoliths again upon re-entry. Such marked changes in otolith input initially produce a mismatch between the brain’s expectation of sensory consequence of head motion and that which is experienced. Accordingly, many flight-based experiments have investigated how the vestibular sensory organs as well as central vestibular neural pathways respond and adapt to the transition from gravity to microgravity and *vice versa*. For example, increased otoconial mass and hair cell numbers are observed following a week in microgravity, as are central changes in the brainstem and cerebellum. Correspondingly, opposite trends are observed upon re-entry.

Importantly, such space-based investigation has been furthered by single unit studies using ground-based models—including labyrinthectomy and sensorimotor learning—in which the relationship between expected and actual vestibular input is systematically altered. The results of these ground-based experiments suggest that the brain uses two key strategies to adapt to altered gravity: (i) the updating of a cerebellum-based internal model of the sensory consequences of gravity; and (ii) the re-weighting of extra-vestibular information as the vestibular system becomes less (i.e., entering microgravity) and then again more reliable (i.e., return to earth). Both strategies have rapid time courses, with the updating of a cerebellum-based model of the sensory consequences of gravity occurring over a few movements (Brooks et al., 2015; Mackrous et al., 2019), and significant sensory re-weighting occurring within 24 h and stabilizing after ~5 days (Sadeghi et al., 2010, 2011, 2012). Strikingly, it is during this time window that astronauts display motion sickness (reviewed in Carriot et al., 2015). Accordingly, we propose that further advancing our knowledge of the neural mechanisms that mediate adaptation will have important implications for understanding how to optimize training programs that account for the environmental challenges of astronauts before and after space exploration missions. Finally, we note that multiple factors (e.g., changes in plasma volume, heart rate, maximal muscle power, etc.) in addition to altered vestibular input ultimately contribute to impact crew performance during space flight. Understanding the interactions between changes in vestibular input and these additional stressors and their impact on astronaut performance will be an important direction for future research.

## Author Contributions

JC, KC, and IM: conceptualization and writing of the manuscript. All authors contributed to the article and approved the submitted version.

## Conflict of Interest

The authors declare that the research was conducted in the absence of any commercial or financial relationships that could be construed as a potential conflict of interest.

## Publisher’s Note

All claims expressed in this article are solely those of the authors and do not necessarily represent those of their affiliated organizations, or those of the publisher, the editors and the reviewers. Any product that may be evaluated in this article, or claim that may be made by its manufacturer, is not guaranteed or endorsed by the publisher.

## References

[B1] Andreoni G. Rigotti C. Baroni G. Ferrigno G. Colford N. A. Pedotti A. (2000). Quantitative analysis of neutral body posture in prolonged microgravity. Gait Posture 12, 235–242. 10.1016/s0966-6362(00)00088-6 11154934

[B2] Angelaki D. E. (2021). “An internal model of gravity and its role in action, perception and spatial orientation,” in Vestibular-Oriented Research Meeting, (Collumbus, OH: Ohio State University).

[B3] Angelaki D. E. Cullen K. E. (2008). Vestibular system: the many facets of a multimodal sense. Annu. Rev. Neurosci. 31, 125–150. 10.1146/annurev.neuro.31.060407.125555 18338968

[B4] Anken R. H. Kappel T. Rahmann H. (1998). Morphometry of fish inner ear otoliths after development at 3g hypergravity. Acta Otolaryngol. 118, 534–539. 10.1080/00016489850154685 9726679

[B5] Badakva A. M. Zalkind D. V. Miller N. V. Sirota M. G. Beloozerova I. N. Kozlovskaya I. B. (1993). “Chapter 4. Effects of microgravity on neuronal circuitry involved in forming of the vestibular and oculomotor responses: results of primate studies in BION projects,” in The Final Report on Cosmos 2229 Experiments, (Moscow: Russian Academy of Science), 92–104.

[B6] Baroni G. Pedrocchi A. Ferrigno G. Massion J. Pedotti A. (2001). Motor coordination in weightless conditions revealed by long-term microgravity adaptation. Acta Astronaut 49, 199–213. 10.1016/s0094-5765(01)00099-6 11669110

[B7] Bloomberg J. J. Mulavara A. P. (2003). Changes in walking strategies after spaceflight. IEEE Eng. Med. Biol. Mag. 22, 58–62. 10.1109/memb.2003.1195697 12733460

[B8] Bloomberg J. J. Peters B. T. Smith S. L. Huebner W. P. Reschke M. F. (1997). Locomotor head-trunk coordination strategies following space flight. J. Vestib. Res. 7, 161–177. 10.3233/ves-1997-72-307 9178222

[B10] Boyle R. Mensinger A. F. Yoshida K. Usui S. Intravaia A. Tricas T.. (2001). Neural readaptation to Earth’s gravity following return from space. J. Neurophysiol. 86, 2118–2122. 10.1152/jn.2001.86.4.2118 11600668

[B9] Boyle R. Varelas J. (2021). Otoconia structure after short- and long-duration exposure to altered gravity. J. Assoc. Res. Otolaryngol. 22, 509–525. 10.1007/s10162-021-00791-6 34008038 PMC8476704

[B11] Bracchi F. Gualierotti T. Morabito A. Rocca E. (1975). Multiday recordings from the primary neurons of the statoreceptors of the labyrinth of the bull frog. The effect of an extended period of “weightlessness” on the rate of firing at rest and in response to stimulation by brief periods of centrifugation (OFO-A orbiting experiment). Acta Otolaryngol. Suppl. 334, 1–27. 1082707

[B12] Bringoux L. Blouin J. Coyle T. Ruget H. Mouchnino L. (2012). Effect of gravity-like torque on goal-directed arm movements in microgravity. J. Neurophysiol. 107, 2541–2548. 10.1152/jn.00364.2011 22298835

[B14] Brooks J. X. Carriot J. Cullen K. E. (2015). Learning to expect the unexpected: rapid updating in primate cerebellum during voluntary self-motion. Nat. Neurosci. 18, 1310–1317. 10.1038/nn.4077 26237366 PMC6102711

[B500] Brooks J. X. Cullen K. E. (2009). Multimodal integration in rostral fastigial nucleus provides an estimate of body movement. J. Neurosci. 29, 10499–10511. 10.1523/JNEUROSCI.1937-09.2009 19710303 PMC3311469

[B13] Brooks J. X. Cullen K. E. (2014). Early vestibular processing does not discriminate active from passive self-motion if there is a discrepancy between predicted and actual proprioceptive feedback. J. Neurophysiol. 111, 2465–2478. 10.1152/jn.00600.2013 24671531 PMC4044434

[B15] Carriot J. Bringoux L. Charles C. Mars F. Nougier V. Cian C. (2004). Perceived body orientation in microgravity: effects of prior experience and pressure under the feet. Aviat. Space Environ. Med. 75, 795–799. 10.1523/JNEUROSCI.0692-14.2014 15460632

[B16] Carriot J. Jamali M. Cullen K. E. (2015). Rapid adaptation of multisensory integration in vestibular pathways. Front. Syst. Neurosci. 9:59. 10.3389/fnsys.2015.00059 25932009 PMC4399207

[B17] Cebolla A. M. Petieau M. Dan B. Balazs L. Mcintyre J. Cheron G. (2016). Cerebellar contribution to visuo-attentional alpha rhythm: insights from weightlessness. Sci. Rep. 6:37824. 10.1038/srep37824 27883068 PMC5121637

[B18] Chen Y.-S. Zhou S. (2011). Soleus H-reflex and its relation to static postural control. Gait Posture 33, 169–178. 10.1016/j.gaitpost.2010.12.008 21211976

[B501] Clark J. B. Bacal K. (2008). “Neurologic concerns,” in Principles of Clinical Medicine for Space Flight, eds Barratt M. R. Pool S. L. (New York, NY: Springer), 361–380. 10.1007/978-0-387-68164-1_17

[B19] Clément G. (2018). Perception of time in microgravity and hypergravity during parabolic flight. Neuroreport 29, 247–251. 10.1097/WNR.0000000000000923 29112678

[B23] Clément G. R. Boyle R. D. George K. A. Nelson G. A. Reschke M. F. Williams T. J.. (2020). Challenges to the central nervous system during human spaceflight missions to Mars. J. Neurophysiol. 123, 2037–2063. 10.1152/jn.00476.2019 32292116

[B21] Clément G. Gurfinkel V. S. Lestienne F. Lipshits M. I. Popov K. E. (1984). Adaptation of postural control to weightlessness. Exp. Brain Res. 57, 61–72. 10.1007/BF00231132 6519230

[B22] Clément G. Moore S. T. Raphan T. Cohen B. (2001). Perception of tilt (somatogravic illusion) in response to sustained linear acceleration during space flight. Exp. Brain Res. 138, 410–418. 10.1007/s002210100706 11465738

[B20] Clément G. Wood S. J. (2014). Rocking or rolling—perception of ambiguous motion after returning from space. PLoS One 9:e111107. 10.1371/journal.pone.0111107 25354042 PMC4213005

[B24] Cohen B. Yakushin S. B. Holstein G. R. Dai M. Tomko D. L. Badakva A. M.. (2005). Vestibular experiments in space. Adv. Space Biol. Med. 10, 105–164. 10.1016/s1569-2574(05)10005-7 16101106 PMC12969068

[B25] Cordo P. J. Nashner L. M. (1982). Properties of postural adjustments associated with rapid arm movements. J. Neurophysiol. 47, 287–302. 10.1152/jn.1982.47.2.287 7062101

[B26] Correia M. J. Perachio A. A. Dickman J. D. Kozlovskaya I. B. Sirota M. G. Yakushin S. B.. (1992). Changes in monkey horizontal semicircular canal afferent responses after spaceflight. J. Appl. Physiol. 73, 112S–120S. 10.1152/jappl.1992.73.2.S112 1326513

[B27] Courtine G. Pozzo T. (2004). Recovery of the locomotor function after prolonged microgravity exposure. I. Head-trunk movement and locomotor equilibrium during various tasks. Exp. Brain Res. 158, 86–99. 10.1007/s00221-004-1877-2 15164151

[B28] Crevecoeur F. Thonnard J.-L. Lefevre P. (2010). Sensorimotor mapping for anticipatory grip force modulation. J. Neurophysiol. 104, 1401–1408. 10.1152/jn.00114.2010 20573975

[B29] Cullen K. E. (2019). Vestibular processing during natural self-motion: implications for perception and action. Nat. Rev. Neurosci. 20, 346–363. 10.1038/s41583-019-0153-1 30914780 PMC6611162

[B30] Cullen K. E. Brooks J. X. (2015). Neural correlates of sensory prediction errors in monkeys: evidence for internal models of voluntary self-motion in the cerebellum. Cerebellum 14, 31–34. 10.1007/s12311-014-0608-x 25287644 PMC4320652

[B31] Cullen K. E. Wei R.-H. (2021). Differences in the structure and function of the vestibular efferent system among vertebrates. Front. Neurosci. 15:684800. 10.3389/fnins.2021.684800 34248486 PMC8260987

[B32] Dale A. Cullen K. E. (2015). Local population synchrony and the encoding of eye position in the primate neural integrator. J. Neurosci. 35, 4287–4295. 10.1523/JNEUROSCI.4253-14.2015 25762675 PMC6605287

[B33] Forbes P. A. Vlutters M. Dakin C. J. Van Der Kooij H. Blouin J. S. Schouten A. C. (2017). Rapid limb-specific modulation of vestibular contributions to ankle muscle activity during locomotion. J. Physiol. 595, 2175–2195. 10.1113/JP272614 28008621 PMC5350434

[B34] Gaveau J. Berret B. Angelaki D. E. Papaxanthis C. (2016). Direction-dependent arm kinematics reveal optimal integration of gravity cues. eLife 5:e16394. 10.7554/eLife.16394 27805566 PMC5117856

[B35] Glasauer S. Amorim M. A. Bloomberg J. J. Reschke M. F. Peters B. T. Smith S. L.. (1995). Spatial orientation during locomotion [correction of locomation] following space flight. Acta Astronaut 36, 423–431. 10.1016/0094-5765(95)00127-1 11540973

[B36] Goldberg J. M. Wilson V. J. Angelaki D. E. Cullen K. E. Fukushima K. Buttner-Ennever J. (2012). The Vestibular System: A Sixth Sense. New York, NY: Oxford University Press.

[B37] Gualtierotti T. (1977). The vestibular function research programme as a part of the Spacelab project: an investigation of the effect of free fall on unitary and integrated vestibular activity. Proc. R. Soc. Lond. B Biol. Sci. 199, 493–503. 10.1098/rspb.1977.0157 23543

[B38] Gualtierotti T. Alltucker D. S. (1967). Prolonged recording from single vestibular units in the frog during plane and space flight, its significance and technique. Aerosp. Med. 38, 513–517. 4382550

[B39] Gualtierotti T. Bailey P. (1968). A neutral buoyancy micro-electrode for prolonged recording from single nerve units. Electroencephalogr. Clin. Neurophysiol. 25, 77–81. 10.1016/0013-4694(68)90090-4 4174787

[B40] Hallgren E. Kornilova L. Fransen E. Glukhikh D. Moore S. T. Clement G.. (2016). Decreased otolith-mediated vestibular response in 25 astronauts induced by long-duration spaceflight. J. Neurophysiol. 115, 3045–3051. 10.1152/jn.00065.2016 27009158 PMC4922620

[B41] Han Kim K. Young K. S. Rajulu S. L. (2019). “Neutral body posture in spaceflight,” in Proceedings of the Human Factors and Ergonomics Society Annual Meeting, (Los Angeles, CA: SAGE Publications), 992–996.

[B42] Holstein G. R. Kukielka E. Martinelli G. P. (1999). Anatomical observations of the rat cerebellar nodulus after 24 hr of spaceflight. J. Gravit. Physiol. 6, P47–50. 11543023

[B43] Honeine J.-L. Schieppati M. Gagey O. Do M.-C. (2013). The functional role of the triceps surae muscle during human locomotion. PLoS One 8:e52943. 10.1371/journal.pone.0052943 23341916 PMC3547017

[B44] Honeine J.-L. Schieppati M. Gagey O. Do M.-C. (2014). By counteracting gravity, triceps surae sets both kinematics and kinetics of gait. Physiol. Rep. 2:e00229. 10.1002/phy2.229 24744898 PMC3966244

[B45] Howard I. P. Hu G. (2001). Visually induced reorientation illusions. Perception 30, 583–600. 10.1068/p3106 11430243

[B46] Hupfeld K. E. Mcgregor H. R. Koppelmans V. Beltran N. E. Kofman I. S. De Dios Y. E.. (2021). Brain and behavioral evidence for reweighting of vestibular inputs with long-duration spaceflight. Cereb. Cortex [Epub ahead of print]. 10.1093/cercor/bhab239 34416764 PMC8841601

[B47] Jain V. Wood S. J. Feiveson A. H. Black F. O. Paloski W. H. (2010). Diagnostic accuracy of dynamic posturography testing after short-duration spaceflight. Aviat. Space Environ. Med. 81, 625–631. 10.3357/asem.2710.2010 20597240

[B502] Jamali M. Carriot J. Chacron M. J. Cullen K. E. (2019). Coding strategies in the otolith system differ for translational head motion vs. static orientation relative to gravity. eLife 8:e45573. 10.7554/eLife.45573 31199243 PMC6590985

[B48] Jamali M. Mitchell D. E. Dale A. Carriot J. Sadeghi S. G. Cullen K. E. (2014). Neuronal detection thresholds during vestibular compensation: contributions of response variability and sensory substitution. J. Physiol. 592, 1565–1580. 10.1113/jphysiol.2013.267534 24366259 PMC3979612

[B49] Jansen K. De Groote F. Duysens J. Jonkers I. (2014). How gravity and muscle action control mediolateral center of mass excursion during slow walking: a simulation study. Gait Posture 39, 91–97. 10.1016/j.gaitpost.2013.06.004 23816462

[B50] Jenkin H. L. Dyde R. T. Jenkin M. R. Howard I. P. Harris L. R. (2003). Relative role of visual and non-visual cues in determining the direction of “up”: experiments in the York tilted room facility. J. Vestib. Res. 13, 287–293. 10.3233/VES-2003-134-613 15096672

[B52] Kaufman G. D. Anderson J. H. Beitz A. J. (1992a). Brainstem Fos expression following acute unilateral labyrinthectomy in the rat. Neuroreport 3, 829–832. 10.1097/00001756-199210000-00002 1421082

[B53] Kaufman G. D. Anderson J. H. Beitz A. J. (1992b). Fos-defined activity in rat brainstem following centripetal acceleration. J. Neurosci. 12, 4489–4500. 10.1523/JNEUROSCI.12-11-04489.1992 1432106 PMC6576004

[B51] Kaufman G. D. Perachio A. A. (1994). Translabyrinth electrical stimulation for the induction of immediate-early genes in the gerbil brainstem. Brain Res. 646, 345–350. 10.1016/0006-8993(94)90104-x 8069688

[B54] Kozlovskaya I. B. Ilyin E. A. Sirota M. G. Korolkov V. I. Babayev B. M. Beloozerova I. N.. (1989). Studies of space adaptation syndrome in experiments on primates performed on board of Soviet biosatellite “Cosmos-1887”. Physiologist 32, 45–48. 2498915

[B56] Kozlovskaya I. B. Sirota M. G. Beloozerova I. N. Yakushin S. B. (1991). “Vestibulo-motor interaction in microgravity,” in International Meeting “Biosatellite Cosmos” (Leningrad, Moscow).

[B55] Kozlovskaya I. B. Sirota M. B. Yakushin S. B. Beloozerova I. I. Babayev B. M. Badakva A. M.. (1994). “The influence of weightlessness on parameters of the horizontal vestibulo-ocular reflex (HVOR) in monkeys,” in Space Biology and Aviacosmic Medicine. Report Abstracts of X Conference (Slovo, Moscow), 113–114.

[B57] Krasnov I. B. (1991). The otolith apparatus and cerebellar nodulus in rats developed under 2-G gravity. Physiologist 34, S206–207. 2047444

[B58] Krasnov I. B. D’iachkova L. N. (1986). [Ultrastructure of the cortex of the cerebellar nodulus in rats after a flight on the biosatellite Kosmos-1514]. Kosm. Biol. Aviakosm. Med. 20, 45–48. 3784524

[B59] Krasnov I. B. Dyachkova L. N. (1990). The effect of space flight on the ultrastructure of the rat cerebellar and hemisphere cortex. Physiologist 33, S29–30. 2371337

[B60] Lackner J. R. Dizio P. (1994). Rapid adaptation to Coriolis force perturbations of arm trajectory. J. Neurophysiol. 72, 299–313. 10.1152/jn.1994.72.1.299 7965013

[B61] Lackner J. R. DiZio P. (2000). Human orientation and movement control in weightless and artificial gravity environments. Exp. Brain Res. 130, 2–26. 10.1007/s002210050002 10638437

[B62] Lackner J. R. DiZio P. (2005). Motor control and learning in altered dynamic environments. Curr. Opin. Neurobiol. 15, 653–659. 10.1016/j.conb.2005.10.012 16271464

[B63] Lackner J. R. Dizio P. (2006). Space motion sickness. Exp. Brain Res. 175, 377–399. 10.1007/s00221-006-0697-y 17021896

[B64] Lackner J. R. Graybiel A. (1979). Parabolic flight: loss of sense of orientation. Science 206, 1105–1108. 10.1126/science.493998 493998

[B65] Lacquaniti F. Bosco G. Gravano S. Indovina I. La Scaleia B. Maffei V.. (2014). Multisensory integration and internal models for sensing gravity effects in primates. Biomed. Res. Int. 2014:615854. 10.1155/2014/615854 25061610 PMC4100343

[B66] Lychakov D. V. Pashchinin A. N. Boiadzhieva-Mikhailova A. Khristov I. (1989). [Study of the structure of receptor organs of the vestibular apparatus of rats after space flight on “Kosmos-1667”]. Kosm. Biol. Aviakosm. Med. 23, 17–26. 2593603

[B67] Macaluso T. Bourdin C. Buloup F. Mille M. L. Sainton P. Sarlegna F. R.. (2017). Sensorimotor reorganizations of Arm kinematics and postural strategy for functional whole-body reaching movements in microgravity. Front. Physiol. 8:821. 10.3389/fphys.2017.00821 29104544 PMC5654841

[B68] Mackrous I. Carriot J. Jamali M. Cullen K. E. (2019). Cerebellar prediction of the dynamic sensory consequences of gravity. Curr. Biol. 29, 2698–2710.e4. 10.1016/j.cub.2019.07.006 31378613 PMC6702062

[B69] Marlinski V. McCrea R. A. (2008). Activity of ventroposterior thalamus neurons during rotation and translation in the horizontal plane in the alert squirrel monkey. J. Neurophysiol. 99, 2533–2545. 10.1152/jn.00761.2007 18337373

[B70] Marsden J. F. Blakey G. Day B. L. (2003). Modulation of human vestibular-evoked postural responses by alterations in load. J. Physiol. 548, 949–953. 10.1113/jphysiol.2002.029991 12626679 PMC2342893

[B71] Massion J. Popov K. Fabre J. C. Rage P. Gurfinkel V. (1997). Is the erect posture in microgravity based on the control of trunk orientation or center of mass position? Exp. Brain Res. 114, 384–389. 10.1007/pl00005647 9166928

[B72] Massot C. Chacron M. J. Cullen K. E. (2011). Information transmission and detection thresholds in the vestibular nuclei: single neurons vs. population encoding. J. Neurophysiol. 105, 1798–1814. 10.1152/jn.00910.2010 21307329 PMC3774568

[B73] McIntyre J. Berthoz A. Lacquaniti F. (1998). Reference frames and internal models for visuo-manual coordination: what can we learn from microgravity experiments? Brain Res. Brain Res. Rev. 28, 143–154. 10.1016/s0165-0173(98)00034-4 9795191

[B74] Moore S. T. MacDougall H. G. Peters B. T. Bloomberg J. J. Curthoys I. S. Cohen H. S. (2006). Modeling locomotor dysfunction following spaceflight with Galvanic vestibular stimulation. Exp. Brain Res. 174, 647–659. 10.1007/s00221-006-0528-1 16763834

[B75] Mouchnino L. Cincera M. Fabre J. C. Assaiante C. Amblard B. Pedotti A.. (1996). Is the regulation of the center of mass maintained during leg movement under microgravity conditions? J. Neurophysiol. 76, 1212–1223. 10.1152/jn.1996.76.2.1212 8871231

[B76] Mulavara A. P. Feiveson A. H. Fiedler J. Cohen H. Peters B. T. Miller C.. (2010). Locomotor function after long-duration space flight: effects and motor learning during recovery. Exp. Brain Res. 202, 649–659. 10.1007/s00221-010-2171-0 20135100

[B77] Mulavara A. P. Ruttley T. Cohen H. S. Peters B. T. Miller C. Brady R.. (2012). Vestibular-somatosensory convergence in head movement control during locomotion after long-duration space flight. J. Vestib. Res. 22, 153–166. 10.3233/VES-2011-0435 23000615

[B78] Oman C. M. Cullen K. E. (2014). Brainstem processing of vestibular sensory exafference: implications for motion sickness etiology. Exp. Brain Res. 232, 2483–2492. 10.1007/s00221-014-3973-2 24838552 PMC4130651

[B79] Paloski W. H. Reschke M. F. Black F. O. Doxey D. D. Harm D. L. (1992). Recovery of postural equilibrium control following spaceflight. Ann. N Y Acad. Sci. 656, 747–754. 10.1111/j.1749-6632.1992.tb25253.x 1599180

[B80] Panic H. Panic A. S. Dizio P. Lackner J. R. (2015). Direction of balance and perception of the upright are perceptually dissociable. J. Neurophysiol. 113, 3600–3609. 10.1152/jn.00737.2014 25761954 PMC4461880

[B81] Papaxanthis C. Pozzo T. Popov K. E. Mcintyre J. (1998). Hand trajectories of vertical arm movements in one-G and zero-G environments. Evidence for a central representation of gravitational force. Exp. Brain Res. 120, 496–502. 10.1007/s002210050423 9655235

[B82] Pedrozo H. A. Wiederhold M. L. (1994). Effects of hypergravity on statocyst development in embryonic Aplysia californica. Hear. Res. 79, 137–146. 10.1016/0378-5955(94)90135-x 7806476

[B83] Pompeiano O. d’Ascanio P. Balaban E. Centini C. Pompeiano M. (2004). Gene expression in autonomic areas of the medulla and the central nucleus of the amygdala in rats during and after space flight. Neuroscience 124, 53–69. 10.1016/j.neuroscience.2003.09.027 14960339

[B84] Pompeiano O. d’Ascanio P. Centini C. Pompeiano M. Balaban E. (2002). Gene expression in rat vestibular and reticular structures during and after space flight. Neuroscience 114, 135–155. 10.1016/s0306-4522(02)00202-6 12207961

[B85] Reschke M. F. Anderson D. J. Homick J. L. (1984). Vestibulospinal reflexes as a function of microgravity. Science 225, 212–214. 10.1126/science.6729475 6729475

[B86] Reschke M. F. Bloomberg J. J. Harm D. L. Paloski W. H. Layne C. Mcdonald V. (1998). Posture, locomotion, spatial orientation, and motion sickness as a function of space flight. Brain Res. Brain Res. Rev. 28, 102–117. 10.1016/s0165-0173(98)00031-9 9795167

[B87] Reschke M. F. Wood S. J. Clément G. (2018). Ocular counter rolling in astronauts after short- and long-duration spaceflight. Sci. Rep. 8:7747. 10.1038/s41598-018-26159-0 29773841 PMC5958131

[B88] Ross H. E. Schwartz E. Emmerson P. (1987). The nature of sensorimotor adaptation to altered G-levels: evidence from mass discrimination. Aviat. Space Environ. Med. 58, A148–152. 3675482

[B89] Ross M. D. (1993). Morphological changes in rat vestibular system following weightlessness. J. Vestib. Res. 3, 241–251. 10.3233/VES-1993-3305 7903895

[B90] Ross M. D. (1994). A spaceflight study of synaptic plasticity in adult rat vestibular maculas. Acta Otolaryngol. Suppl. 516, 1–14. 7976320

[B91] Ross M. D. (2000). Changes in ribbon synapses and rough endoplasmic reticulum of rat utricular macular hair cells in weightlessness. Acta Otolaryngol. 120, 490–499. 10.1080/000164800750045983 10958400

[B93] Ross M. D. Donovan K. Chee O. (1985). Otoconial morphology in space-flown rats. Physiologist 28, S219–220. 3834471

[B92] Ross M. D. Tomko D. L. (1998). Effect of gravity on vestibular neural development. Brain Res. Brain Res. Rev. 28, 44–51. 10.1016/s0165-0173(98)00025-3 9795127

[B94] Sadeghi S. G. Minor L. B. Cullen K. E. (2007). Response of vestibular-nerve afferents to active and passive rotations under normal conditions and after unilateral labyrinthectomy. J. Neurophysiol. 97, 1503–1514. 10.1152/jn.00829.2006 17122313

[B95] Sadeghi S. G. Minor L. B. Cullen K. E. (2010). Neural correlates of motor learning in the vestibulo-ocular reflex: dynamic regulation of multimodal integration in the macaque vestibular system. J. Neurosci. 30, 10158–10168. 10.1523/JNEUROSCI.1368-10.2010 20668199 PMC2933842

[B96] Sadeghi S. G. Minor L. B. Cullen K. E. (2011). Multimodal integration after unilateral labyrinthine lesion: single vestibular nuclei neuron responses and implications for postural compensation. J. Neurophysiol. 105, 661–673. 10.1152/jn.00788.2010 21148096 PMC3059170

[B97] Sadeghi S. G. Minor L. B. Cullen K. E. (2012). Neural correlates of sensory substitution in vestibular pathways following complete vestibular loss. J. Neurosci. 32, 14685–14695. 10.1523/JNEUROSCI.2493-12.2012 23077054 PMC3503523

[B98] Scotto Di Cesare C. Sarlegna F. R. Bourdin C. Mestre D. R. Bringoux L. (2014). Combined influence of visual scene and body tilt on arm pointing movements: gravity matters!. PLoS One 9:e99866. 10.1371/journal.pone.0099866 24925371 PMC4055731

[B99] Shipov A. A. Sirota M. G. Beloozerova I. N. Babaev B. M. Kozlovskaya I. B. (1986). “Results of tests on the primate vestibulo-visualmotor reactions in biocosmos experiments,” in Adaptive Processes in Visual and Oculomotor Systems, eds Keller E. L. Zee D. S. (New York, NY: Pergamon Press), 129–132.

[B100] Sirota M. (1988). Neuronal activity of vestibular nuclei during coordinated movement of eyes and head in microgravitation. Physiologist 31, 8–9.

[B101] Sirota M. G. Babayev B. M. Beloozerova I. B. Nyrova A. N. Yakushin S. B. Kozlovskaya I. B. (1987). Characteristics of vestibular reactions to canal and otolith stimulation at an early stage of exposure to microgravity. Physiologist 30, S82–84. 3104942

[B102] Sirota M. G. Babayev B. M. Beloozerova I. N. Nyrova A. N. Yakushin S. B. Kozlovskaya I. B. (1988a). Neuronal activity of nucleus vestibularis during coordinated movement of eyes and head in microgravitation. Physiologist 31, 8–9.

[B106] Sirota M. G. Beloozerova I. N. Babaev B. M. Yakushin S. B. Ivanov A. M. Nyrova A. N.. (1988b). “Chapter 7.2. The vestibular function and vestibulo-oculomotor interactions in microgravity,” in The Final Report on Cosmos 1887 Experiments (Moscow: Ministry of Health of the USSR), 1–50.

[B103] Sirota M. G. Beloozerova I. N. Babaev B. M. Kozlovskaya I. B. (1984). “Chapter 2.4. Study of the vestibular function and vestibulo-oculomotor interaction in monkey,” in The Final Report on Cosmos 1514 Experiments, (Moscow: Ministry of Health of the USSR), 1–31.

[B105] Sirota M. G. Beloozerova I. N. Babaev B. M. Yakushin S. B. Ivanov A. M. Kozlovskaya I. B. (1986). “Chapter 7. The vestibular function and vestibulo-oculomotor interaction in microgravity,” in The Final Report on Cosmos 1667 Experiments, (Moscow: Ministry of Health of the USSR), 119–162.

[B104] Sirota M. G. Beloozerova I. N. Babaev B. M. Yakushin S. B. Kozlovskaya I. B. (1989a). “Effects of microgravity on vestibular function in monkey. Results of space flight studies,” in Eleventh Annual Meeting IUPS Commission on Gravitational Physiology (Lyon, France).

[B107] Sirota M. G. Beloozerova I. N. Yakushin S. B. Kozlovskaya I. B. (1989b). “Eye-head coordination during adaptation to microgravity,” in Regulation of Sensor-Motor Functions (Vinniza, USSR).

[B108] Sirota M. G. Beloozerova I. N. Yakushin S. B. Babaev B. M. Kozlovskaya I. B. (1990a). “Changes in neuronal activity of vestibular nuclei during active head movements in microgravity,” in XII International Meeting of Gravitational Physiology (Leningrad, Moscow).

[B109] Sirota M. G. Beloozerova I. N. Yakushin S. B. Babaev B. M. Kozlovskaya I. B. (1990b). “Influence of microgravity to vestibular function of monkey,” in XII International Meeting of Gravitational Physiology (Leningrad, Moscow), 63–64.

[B110] Sirota M. G. Beloozerova I. N. Yakushin S. B. Babaev B. M. Kozlovskaya I. B. (1990c). “Vestibulo-motor interaction of monkey in microgravity. Eye-head coordination test,” in XI Meeting in Space Biology and Medicine (Kaluga, Moscow), 342–343.

[B113] Sirota M. G. Beloozerova I. N. Yakushin S. B. Ivanov A. M. Kozlovskaya I. B. (1991c). “Kinematic of saccades and head movement in eye-head coordination test of monkey. Experiment on biosatellite “Cosmos-2044” board,” in International Meeting “Biosatellite Cosmos” (Leningrad, Moscow).

[B111] Sirota M. G. Beloozerova I. N. Yakushin S. B. Babaev B. M. Kozlovskaya I. B. (1991a). “Chapter 6. Effects of the space flight on primate’s motor and vestibular functions,” in The Final Report on Cosmos 2044 Experiments (Moscow: Ministry of Health of the USSR), 223–295.

[B112] Sirota M. G. Beloozerova I. N. Babaev B. M. Yakushin S. B. Kozlovskaya I. B. (1991b). “Gain of vestibulo-ocular reflex of monkey in microgravity. Experiment on biosatellite “Cosmos-2044” board,” in International Meeting “Biosatellite Cosmos” (Leningrad, Moscow).

[B114] Souvestre P. A. Landrock C. K. Blaber A. P. (2008). Reducing incapacitating symptoms during space flight: is postural deficiency syndrome an applicable model? Hippokratia 12, 41–48. 19048092 PMC2577399

[B115] Sultemeier D. R. Choy K. R. Schweizer F. E. Hoffman L. F. (2017). Spaceflight-induced synaptic modifications within hair cells of the mammalian utricle. J. Neurophysiol. 117, 2163–2178. 10.1152/jn.00240.2016 28228581 PMC5454470

[B116] Sylos-Labini F. Ivanenko Y. P. Cappellini G. Portone A. Maclellan M. J. Lacquaniti F. (2013). Changes of gait kinematics in different simulators of reduced gravity. J. Mot. Behav. 45, 495–505. 10.1080/00222895.2013.833080 24079466

[B117] Vernazza-Martin S. Martin N. Massion J. (2000). Kinematic synergy adaptation to microgravity during forward trunk movement. J. Neurophysiol. 83, 453–464. 10.1152/jn.2000.83.1.453 10634887

[B118] Vinnikov Ia A. Lychakov D. V. Pal’mbakh L. R. Govardovskii V. I. Adanina V. O. (1980). [Vestibular apparatus study of the toad, Xenopus laevis and rats under prolonged weightlessness]. Zh. Evol. Biokhim. Fiziol. 16, 574–579. 6970471

[B119] Watt D. G. Money K. E. Tomi L. M. (1986). M.I.T./Canadian vestibular experiments on the Spacelab-1 mission: 3. Effects of prolonged weightlessness on a human otolith-spinal reflex. Exp. Brain Res. 64, 308–315. 10.1007/BF00237748 3803475

[B120] White O. Gaveau J. Bringoux L. Crevecoeur F. (2020). The gravitational imprint on sensorimotor planning and control. J. Neurophysiol. 124, 4–19. 10.1152/jn.00381.2019 32348686

[B121] Winter D. A. Patla A. E. Prince F. Ishac M. Gielo-Perczak K. (1998). Stiffness control of balance in quiet standing. J. Neurophysiol. 80, 1211–1221. 10.1152/jn.1998.80.3.1211 9744933

[B122] Winter D. A. Prince F. Patla A. (1997). Validity of the inverted pendulum model of balance in quiet standing. Gait Posture 2, 153–154. 10.1016/s0966-6362(97)83376-0 15013500

[B123] Wolpert D. M. Ghahramani Z. (2000). Computational principles of movement neuroscience. Nat. Neurosci. 3, 1212–1217. 10.1038/81497 11127840

[B124] Wolpert D. M. Miall R. C. Kawato M. (1998). Internal models in the cerebellum. Trends Cogn. Sci. 2, 338–347. 10.1016/s1364-6613(98)01221-2 21227230

[B125] Wood S. J. Paloski W. H. Clark J. B. (2015). Assessing sensorimotor function following ISS with computerized dynamic posturography. Aerosp. Med. Hum. Perform. 86, A45–A53. 10.3357/AMHP.EC07.2015 26630195

[B126] Yakusheva T. A. Shaikh A. G. Green A. M. Blazquez P. M. Dickman J. D. Angelaki D. E. (2007). Purkinje cells in posterior cerebellar vermis encode motion in an inertial reference frame. Neuron 54, 973–985. 10.1016/j.neuron.2007.06.003 17582336

[B127] Yakushin S. B. Beloozerova I. N. Sirota M. G. Kozlovskaya I. B. (1989). “Cerebellar and brain stem structures activity during semicircular canals stimulation in microgravity,” in Regulation of Sensomotor Functions, (Vinniza, Moscow, USSR).

[B128] Yakushin S. B. Beloozerova I. N. Sirota M. G. Kozlovskaya I. B. (1990). “Neuronal activity of brain stem vestibular structures and cerebellum of monkey in space flight,” in IX Meeting in Space Biology and Medicine (Vinniza, Moscow).

[B129] Yakushin S. B. Sirota M. G. Beloozerova I. N. Babayev B. M. Kozlovskaya I. B. (1992). “Eye-head coordination of monkey (*Macaca mulatta*) in gaze fixation reaction during “Cosmos” biosatellites flight,” in XVIIth Barany Society Meeting, (Czechoslovakia).

[B130] Zhang J. Peng Z. Yang M. Zhang X. Wei J. Xu M.. (2005). Observation of the morphology and calcium content of vestibular otoconia in rats after simulated weightlessness. Acta Otolaryngol. 125, 1039–1042. 10.1080/00016480510037915 16298783 PMC2858292

[B131] Zupan L. H. Merfeld D. M. Darlot C. (2002). Using sensory weighting to model the influence of canal, otolith and visual cues on spatial orientation and eye movements. Biol. Cybern. 86, 209–230. 10.1007/s00422-001-0290-1 12068787

